# Spatiotemporal characteristics and geophysical drivers of the fractal dimension and *b*-value in North China seismicity

**DOI:** 10.1371/journal.pone.0349558

**Published:** 2026-06-01

**Authors:** Jinmeng Bi, Ye Li, Yuhang Pan

**Affiliations:** 1 Tianjin Earthquake Agency, Tianjin, China; 2 Institute of Geophysics, China Earthquake Administration, Beijing, China; Ministry of Education, MOROCCO

## Abstract

The North China Craton Destruction Zone, one of continental China’s most seismically active regions, exhibits complex spatiotemporal seismicity patterns due to its intricate geology. Using the North China Block earthquake catalog (1970–2025), we applied a stochastic declustering method based on the spatiotemporal Epidemic-Type Aftershock Sequence (ETAS) model to process the data. We then calculated the spatial fractal dimension and the *b*-value of seismicity using the correlation dimension and the maximum likelihood method, respectively. These analyses were conducted to explore their relationships with regional tectonic setting, stress state, and geophysical field characteristics. Declustering increased the spatial fractal dimension of background seismicity from 1.20 to 1.45, effectively reducing spatial clustering without significantly altering the statistical characteristics of the b-value. The fractal dimension is heterogeneous, with high values concentrated in tectonically fragmented zones like the Fen-Wei seismic belt and the central-northern North China Plain, while low values correspond to stable areas such as the Ordos Block. The fractal dimension exhibited systematic temporal changes, characterized by a decreasing before the mainshock and a recovery afterward. This pattern potentially reflects a transition from strain localization, akin to rupture nucleation, to post-seismic stress readjustment, suggesting it may reflect pre-seismic processes. The spatial relationship between fractal dimension and the *b*-value is complex, showing negative correlation in extreme value zones and positive correlation in moderate value zones. Different parameter combinations (*b*-value and fractal dimension) can indicate distinct seismic hazard states. Quantitative analysis reveals the strongest positive correlation between fractal dimension and maximum shear strain rate, and a negative correlation with terrestrial heat flow, confirming that seismic spatial complexity is primarily driven by tectonic forces and modulated by deep thermal state. This study provides refined fractal-based criteria for characterizing seismicity and informing medium- to long-term seismic hazard assessment in the region.

## 1. Introduction

The North China Block, a complex tectonic region shaped by ancient cratonic destruction and intense Cenozoic extension, experiences present-day seismicity jointly controlled by the far-field effects of the westward subduction of the Pacific Plate and the northward compression of the Indian Plate [1]. This dynamic interaction has formed a distinctive basin-and-range tectonic system and its associated seismic activity. Historically, the region has been struck by multiple devastating earthquakes, such as the 1679 Sanhe-Pinggu *M*8.0 and the 1976 Tangshan *M*7.8 events, both causing extensive casualties and economic losses [2].

The *b*-value in the frequency-magnitude relationship is a power-law parameter describing magnitude distribution, while the two-point spatial correlation dimension of earthquake epicenters characterizes the power-law relationship between spatial clustering and randomness of seismic events [3–5]. The *b*-value is commonly linked to stress levels and medium homogeneity along fault zones, it generally decreases with increasing brittle crustal stress, thus indicating shifts in the probability of large (low *b*-value) versus small (high *b*-value) earthquakes [6–8]. In tectonically active regions, *b*-values often deviate from stability, whereas they tend to remain relatively constant over time in stable areas with low seismicity [9,10]. Notably, while the *b*-value associated with individual faults exhibits a degree of universality, the fractal dimension of fault networks is strongly influenced by geological heterogeneity [11–13]. Furthermore, various tectonic processes often preferentially activate fault systems in asperity-rich zones, where most large earthquakes tend to nucleate [14,15]. Consequently, a deeper understanding of the underlying patterns and dynamic mechanisms of seismicity in North China holds significant scientific importance and urgent practical value for disaster prevention and mitigation.

Traditional seismicity studies, largely based on statistical parameters such as the Gutenberg–Richter relationship (*b*-value) and recurrence intervals, can reveal certain statistical regularities but often fall short in effectively characterizing the complexity of spatial structures and the nonlinear features of temporal evolution in seismic activity. The concept of fractal dimension was introduced by Mandelbrot [16] to characterize self-similar geometric objects that are irregular and scale-invariant. Fractal theory, a powerful tool for studying self-similarity in complex systems, offers a new perspective for quantitatively describing the spatiotemporal clustering properties of earthquakes. It has become an important means for analyzing the spatiotemporal complexity of seismic activity, fault activity, stress state, and precursory features [17,18]. The fractal dimension serves as a significant precursory indicator before strong earthquakes; prior to major events, the fractal dimension of earthquake sequences in both time and space often shows anomalous changes, providing potential references for earthquake prediction [19,20]. Multifractal analysis has been widely applied to examine earthquake characteristics across various seismotectonic settings, including the analysis of time series and spatial distributions in the Eurasian seismic belt [21], the investigation of aftershock decay and source parameters following the 2023 Türkiye doublet [22], and the assessment of *b*-value and correlation dimension variations with depth in the central Himalaya [5,13]. Through fractal dimensions, it is possible not only to quantitatively assess fault zone activity and regional seismicity levels but also to effectively characterize the clustering features of seismic events. This, in turn, helps reveal spatial variations in crustal medium anisotropy and tectonic stress distribution, providing a crucial basis for understanding the physical processes of earthquake preparation and conducting hazard analysis.

The relationship between the seismicity parameter *b*-value and the spatial fractal dimension is an important clue for understanding the seismic behavior and potential hazard of different fault zones or tectonic units [23]. Theoretically, regions with low *b*-values (often indicating higher stress levels) and high fractal dimension (reflecting a more uniform spatial distribution of earthquakes) are considered to bear greater tectonic stress and are often associated with higher seismic risk [24–29]. This combination may suggest an area’s capacity for spatially dispersed ruptures under high-stress conditions. However, the coupling between these two parameters varies across different tectonic settings, with studies reporting positive, negative, or insignificant correlations [19,25,28,30–32], highlighting the complexity of their relationship. The non-universal nature of the *b*-value and fractal dimension also indicates their dependence on local geological structures and geophysical conditions.

Nevertheless, systematic research remains lacking on how the *b*-value and fractal dimension are spatially coupled within the specific and complex tectonic context of the North China Craton Destruction Zone, as well as on the deep processes controlling their spatial distribution. This study aims to calculate seismicity parameters (*a*-value, *b*-value) and the spatial fractal dimension before and after declustering using a stochastic method based on the spatiotemporal Epidemic-Type Aftershock Sequence (ETAS) model. We seek to obtain the spatial distribution characteristics of these parameters across seismic zones and seismotectonic units within the North China Block, analyze their spatial variations and coupling relationships, and explore their associations with active faults, strain rate fields, and terrestrial heat flow.

Previous studies have extensively investigated the *b*-value and fractal dimension, yet systematic analyses of their spatial coupling within the specific tectonic context of the North China Craton Destruction Zone remain limited, particularly regarding the deep physical processes governing their distribution. Furthermore, most existing research lacks quantitative correlation between these seismicity parameters and key geophysical fields such as strain rate and terrestrial heat flow. This study addresses these gaps by applying stochastic declustering based on the spatiotemporal ETAS model, mapping the spatial coupling between fractal dimension and *b*-value across tectonic units, and establishing their relationships with maximum shear strain rate and heat flow. This integrated approach identifies dominant physical controls on seismic complexity and provides refined multi-parameter criteria for medium- to long-term hazard assessment.

## 2. Data and methods

### 2.1. Seismic data and tectonic zoning

**Fig 1:**
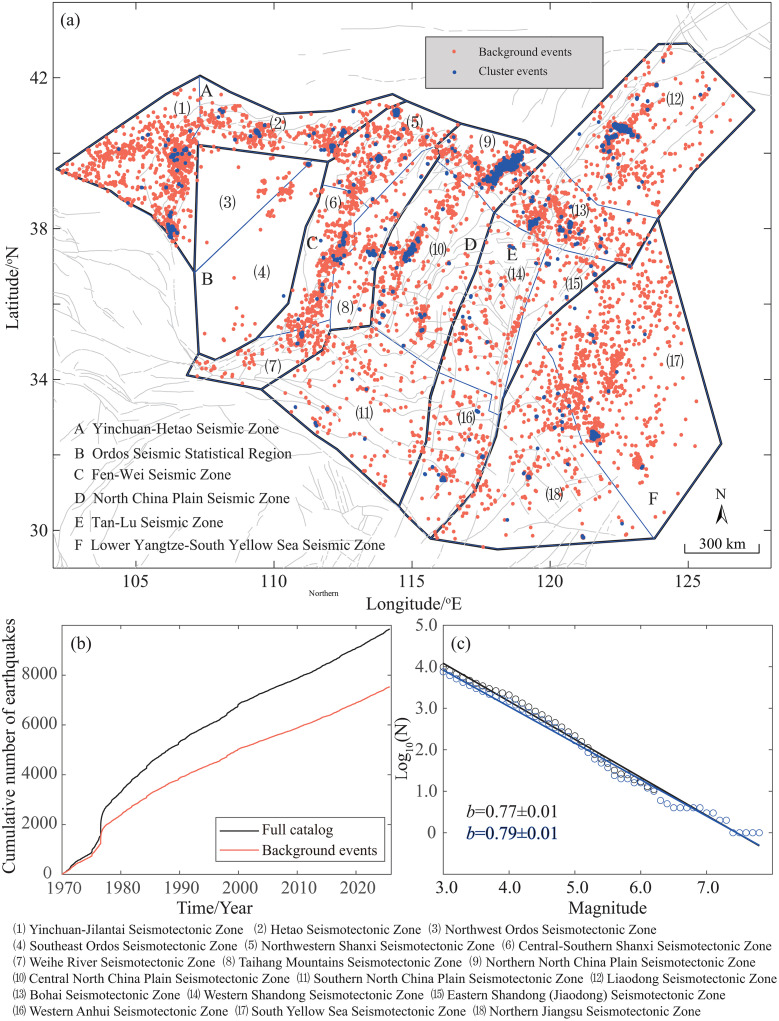
Spatiotemporal characteristics of seismicity in the North China Block. (a) Epicentral distribution of *M* ≥ 3.0 earthquakes from 1 January 1970 to 30 September 2025, color-coded by magnitude, with black lines indicating active faults (b) Cumulative frequency curves for all events and background events after stochastic declustering based on the spatiotemporal ETAS model (c) Magnitude-frequency distribution and fitted *b*-values for all events and background events.

To systematically investigate the seismicity parameters of the North China Block, this study utilizes the National Unified Official Catalogue earthquake catalog provided by the China Earthquake Networks Center (CENC). The catalog covers the period from 1 January 1970–30 September 2025 within the geographical range of 102°E–128°E and 29°N–44°N. During this period, a total of 9,853 earthquakes with *M* ≥ 3.0 were recorded in the North China Block, including 7761 events with *M*3.0-3.9, 1876 events with *M*4.0-4.9, 199 events with *M*5.0-5.9, 14 events with *M*6.0-6.9, and 3 events with *M* ≥ 7.0. Notable among these is the devastating 28 July 1976 Tangshan *M*7.8 earthquake. The spatial distribution of these events is shown in Fig 1.

Considering the regional tectonic framework and the seismotectonic setting, we referred to the zoning scheme of GB 18306–2015 Seismic Ground Motion Parameters Zonation Map of China [33] to subdivide the North China seismic region. It was divided into six seismic zones: the Lower Yangtze-Yellow Sea Seismic Zone, the Tan-Lu Seismic Zone, the North China Plain Seismic Zone, the Fen-Wei Seismic Zone, the Yinchuan-Hetao Seismic Zone, and the Ordos Seismic Statistical Region. Each seismic zone was further subdivided into potential seismic source areas, resulting in a total of 18 such areas, as shown in Fig 1a. Due to its relatively weak and unevenly distributed seismicity, the Ordos Seismic Statistical Region was excluded from subsequent parameter statistical analyses.

### 2.2. Completeness magnitude assessment and stochastic declustering

To ensure the completeness of the dataset throughout the study period and the reliability of the seismicity parameter estimates, a minimum completeness magnitude of *M*_c_ = *M*_L_3.0 was selected for the North China Block. This choice was based on a comprehensive assessment of the dynamic monitoring capability, incorporating both qualitative and quantitative evaluations [34], and takes into account minor fluctuations in the early-stage completeness magnitude. However, considering the impact of the Tangshan earthquake, for which the post-event monitoring capability was assessed as *M*4.0 [35], the completeness magnitude was set to *M*_c_ = *M*_L_4.0 specifically for the North China Plain Seismic Zone and the Northern North China Plain Seismotectonic Zone, which were affected by the Tangshan *M*7.8 event.

To further investigate the characteristics of background seismicity in the North China Block, a stochastic declustering method based on the spatiotemporal ETAS model was applied. This method probabilistically identifies and removes clustered events (mainly aftershocks and swarms), generating a corresponding declustered catalog for comparative analysis with the original catalog. Compared to deterministic time-space window declustering methods, the spatiotemporal ETAS-based approach better preserves coupling characteristics and reduces the underestimation of the background seismicity rate. It demonstrates good adaptability in regions like North China, which experience frequent strong earthquakes and complex aftershock sequences, thereby providing a cleaner tectonically driven earthquake catalog for subsequent seismicity parameter calculation and hazard analysis [2]. The declustering process reduced the number of events from 9853 to 7535 (a declustering rate of 23.53%). Fig 1b shows the cumulative number of all events and declustered events. The temporal clustering of the earthquake sequence was significantly reduced after declustering, with the interevent time distribution becoming more consistent with a Poisson process. This aids in extracting background seismic activity information more directly related to tectonic loading (Supporting Information).

### 2.3. *b-*value estimation and fractal dimension

The spatial fractal dimension effectively quantifies the degree of spatial clustering of earthquakes, thereby revealing the complexity of fault systems and the heterogeneity of crustal media [6]. In contrast, the *b*-value is closely related to material strength and stress state [36,37], it has also been shown that low *b*-value have higher stress accumulation at the laboratory level [38,39]. Both parameters provide valuable insights into earthquake genesis [25,40,41].

The fractal dimension, originally defined by Mandelbrot [16], offers a measure of clustering and material heterogeneity and is commonly derived from earthquake catalogs using methods such as the correlation dimension and box-counting dimension [42–45]. Compared to box-counting, the correlation dimension method has been shown to yield more robust estimates of the fractal dimension [46]. Therefore, in this study we apply the correlation dimension method [47] to estimate the spatial fractal dimension.

The *b*-value is typically calculated using the least-squares method or the maximum likelihood method [36,48,49]. However, the maximum likelihood estimator is generally recognized as more robust and accurate than the least-squares approach [46,50]. Accordingly, in this paper the *b*-value is estimated using the maximum likelihood method, and the spatial fractal dimension is derived from the correlation dimension method.

The *b*-value is estimated using Maximum Likelihood Estimation (MLE) by Aki [51].


$$\begin{array}{c} b=\frac{{log}_{10}\left(e\right)}{\left[\overset{―}{M}-\left(M_{c}-\Delta M_{bin}/2\right)\right]} \end{array}$$ (1)

Here, $\overset{―}{M}$ is the average magnitude of *M* ≥ *M*_c_, $\Delta M_{bin}$ is the magnitude bin of the earthquake catalog, *M*_c_ = 3.0 and △*M*_bin_ = 0.1.

The uncertainty estimation of the *b-*value can be obtained by the following equation [52]:


$$\begin{array}{c} \delta b=2.30b^{\text{2}}\sqrt{\sum_{i=1}^{n}(M_{i}-\overset{―}{M}{)}^{\text{2}}/n(n-1)} \end{array}$$ (2)

where *n* is the number of seismic events.

The fractal dimension of the spatial distribution of seismicity is calculated from the correlation integral given by Grassberger and Procaccia [47] as


$$\begin{array}{c} D_{\text{c}}=\underset{r\to \text{0}}{\text{lim}}\frac{\text{logC}(r)}{\text{log}(r)} \end{array}$$ (3)

where *C*(*r*) is the correlation function that measures the spacing or clustering of a set of points and is given as


$$\begin{array}{c} \text{C}(r)=\frac{\text{2}}{N_{c}(N_{c}-\text{1})}\sum_{i}^{N_{c}}\sum_{i=\;\;/\;j}^{N_{c}}H(r-r_{ij}) \end{array}$$ (4)

where *N*_c_ is the total number of earthquakes, *H*(*r*-*r*_ij_) is the Heaviside step function, *r* is the scaling radius (*r* = 5 km to *r* = 45 km for this study), and *r*_ij_ is the distance between the two epicentres determined by the spherical triangle method [30].

## 3. Results

### 3.1. Temporal evolution of the fractal dimension and *b*-value

**Fig 2:**
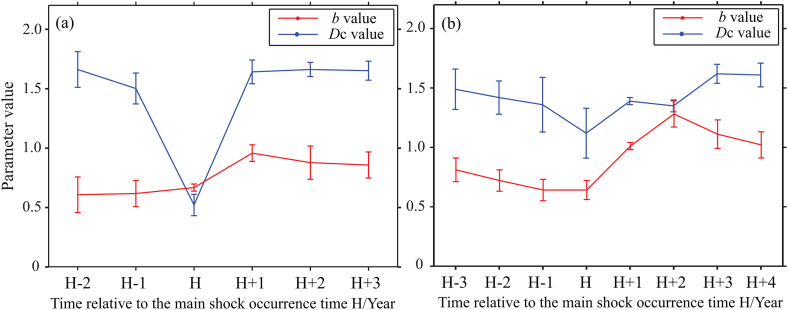
Temporal evolution of fractal dimension *D*_c_ and *b*-value before and after strong earthquakes. (a) The Haicheng *M*_S_7.3 earthquake sequence of 4 February 1975 (b) The Tangshan *M*_S_7.8 earthquake sequence of 28 July 1976. Parameters were calculated using a three-year moving window with a one-year step. H is the origin time of the mainshock.

To examine pre-seismic activity characteristics in relation to aftershock distribution scales and the tectonic features of seismic faults, we selected background events preceding the 4 February 1975 Haicheng *M*_S_7.3 and the 28 July 1976 Tangshan *M*_S_7.8 earthquakes. Due to the significant decline in monitoring capability immediately after the mainshocks, different completeness magnitudes were necessarily adopted for the pre- and post-seismic periods (*M*_L_2.0 pre-seismic, *M*_L_4.0 post-seismic) to maximize event counts. While this adjustment ensures sufficient sample sizes, we acknowledge that it may introduce some uncertainty in comparing absolute values across the mainshock. Therefore, the temporal trends should be interpreted with caution, with emphasis on the relative changes rather than absolute magnitudes. Constrained by the available observational data, we utilized five years of data around the Haicheng event and seven years around the Tangshan event. The temporal variations in the *b*-value and the fractal dimension around the epicentral regions were then analyzed using a moving time window of three years with a one-year step. The results are presented in Fig 2.

A systematic decline in the fractal dimension was observed 5–6 years before the strong earthquakes, with values reaching their minimum approximately three years prior to the mainshocks. This pre-seismic decrease likely reflects an accelerated accumulation of strain and the transition from distributed cracking to localized clustering, indicative of a rupture nucleation process. Following the mainshocks, the fractal dimension gradually recovered, returning to background levels over several years a pattern that mirrors the post-seismic readjustment and redistribution of stress. Correspondingly, the seismicity parameter *b*-value remained relatively low before the mainshocks, signaling progressive stress accumulation. And it then progressively recovered after the mainshocks, consistent with the release of accumulated seismic energy.

For the Haicheng earthquake sequence, the fractal dimension exhibited a slight decreasing trend before the mainshock, followed by a sharp decline to its minimum as the event approached. After the mainshock, it recovered rapidly to approximately pre-seismic levels. The seismicity parameter *b*-value fluctuated within a low range prior to the mainshock, increased sharply immediately after the event, and then gradually decreased. In contrast, for the Tangshan sequence, both the fractal dimension and the *b*-value showed a decreasing trend before the mainshock, reaching their minima around the time of the mainshock. Subsequently, they increased rapidly to high values. Following this peak, the *b*-value decreased to a stable range, while the fractal dimension fluctuated within a certain interval. The fractal dimension and *b*-value show systematic temporal changes around major earthquakes, characterized by pre-seismic decrease and post-seismic recovery, reflecting stress accumulation and release processes.

### 3.2. Spatial distribution characteristics of the fractal dimension and *b*-value

**Fig 3:**
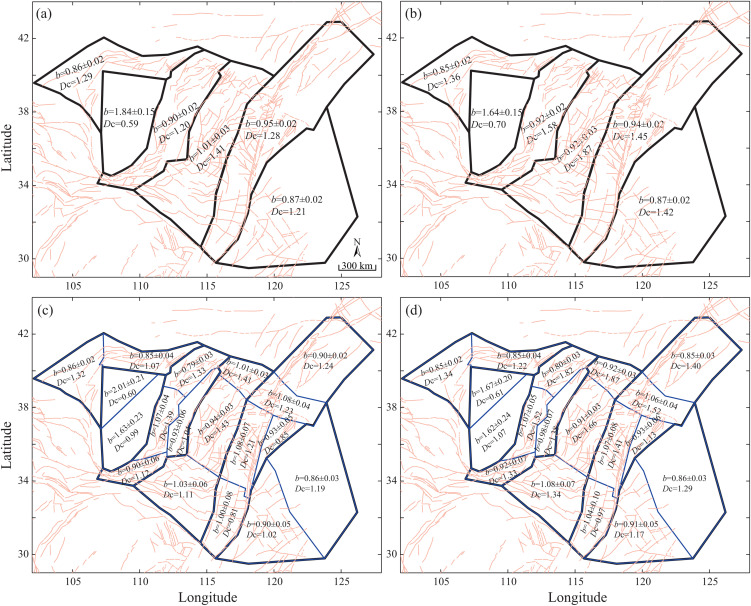
Spatial distribution of the fractal dimension (*D*_c_) and the seismicity parameter *b*-value before and after declustering. Left column: pre-declustering; right column: post-declustering. (a, b) Parameter distribution across seismic zones (c, d) Parameter distribution across potential seismic source zones (seismotectonic zones).

Using the correlation dimension and maximum likelihood methods, we calculated the spatial fractal dimension and seismicity parameters across the North China Block before and after declustering (Fig 3). The overall spatial fractal dimension increased from 1.20 before declustering to 1.45 after declustering. This change indicates that, once the interference from aftershocks and clustered events is removed, the spatial distribution of earthquakes shifts from a relatively concentrated pattern to a more dispersed one. The *a*-value decreased slightly from 6.30 to 6.24, while the *b*-value increased marginally from 0.77 ± 0.01 to 0.79 ± 0.01 (Fig 1c), suggesting minor changes in regional stress or activity patterns. Both parameters exhibit strong spatial heterogeneity across tectonic units.

The spatial fractal dimension of background seismicity in North China is 1.45 overall, indicating that earthquakes in the region exhibit a relatively uniform yet complex spatial distribution. Parameters before and after declustering are summarized for the six seismic zones and the 18 seismotectonic zones, revealing pronounced spatial heterogeneity across these units (Fig 3). Declustering did not significantly alter the *b*-value, however, it increased the fractal dimension for each seismic zone, which aligns with the overall findings.

From a statistical perspective, high *D*_c_ values are concentrated in the Fen-Wei seismic zone and the central-northern North China Plain, regions characterized by Cenozoic fault-depression basins and complex fault intersections. This tectonic setting reflects highly fractured crust that promotes spatially dispersed seismicity. Conversely, low *D*_c_ values occur in tectonically stable regions such as the Ordos Block, the Lower Yangtze-South Yellow Sea seismic zone, and the southern segment of the Tan-Lu fault zone, where simpler geological conditions lead to more clustered earthquakes. Low *b*-values are observed in the Yinchuan-Hetao seismic zone, the Lower Yangtze seismic zone, and parts of the Fen-Wei and North China Plain seismic zones, suggesting elevated stress levels or more intact media, and thus warrant attention for potential moderate-to-strong earthquakes. The spatial pattern of the *b*-value remained stable after declustering.

Except for the Ordos seismic statistical region, the variation in the seismicity parameter *b*-value before and after declustering generally falls within the range of 0–0.09, with minimal fluctuation in the overall mean. This indicates that the stochastic declustering method based on the spatiotemporal ETAS model did not significantly alter the statistical characteristics of the *b*-value. In contrast, the spatial fractal dimension increased markedly after declustering, reflecting a substantial reduction in the clustering of seismic events and a trend toward a more uniform spatial distribution following the removal of clustered sequences. This result demonstrates that the stochastic declustering method effectively reduces spatial clustering effects, thereby more authentically revealing the distribution structure of background seismic activity. By integrating analyses of the spatial fractal dimension and seismicity parameters, this study systematically characterizes the spatiotemporal evolution of seismic activity in North China before and after removing clustering effects and reveals its spatial heterogeneity. This provides a quantitative basis for a deeper understanding of the region’s seismic activity and potential earthquake hazards.

Additionally, this study further analyzed the distribution characteristics of seismicity parameters across sub-regions using quartiles, as detailed in Table 1. The results show that the first (Q1) and third (Q3) quartiles of the *b*-value changed only minimally before and after declustering, shifting from 0.87 to 0.86 and from 1.06 to 1.07, respectively. This indicates that the core distribution range of the *b*-value remained stable and was not significantly affected by the declustering process. In contrast, both quartiles of the fractal dimension increased noticeably after declustering, rising from 1.03 to 1.17 (Q1) and from 1.33 to 1.52 (Q3). This upward shift in the entire distribution signifies that earthquake events became more uniformly distributed in space following declustering. Declustering systematically increases the fractal dimension across all tectonic units, revealing the true spatial complexity of background seismicity, while leaving the *b*-value largely unchanged. High *D*_c_ values concentrate in tectonically fragmented zones, and low values in stable blocks.

**Table 1:** Statistical characteristics of potential seismic source zones (seismotectonic zones) in the North China Block.

		Mean	First Quartile (Q1)	Median	Third Quartile (Q3)
Pre-Declustering	*b-*value	1.01	0.87	0.93	1.06
	*D*_c_ value	1.15	1.03	1.19	1.33
Post-Declustering	*b-*value	1.02	0.86	0.93	1.07
	*D*_c_ value	1.34	1.17	1.34	1.52

### 3.3. Spatial coupling characteristics of fractal dimension and *b*-value

**Fig 4:**
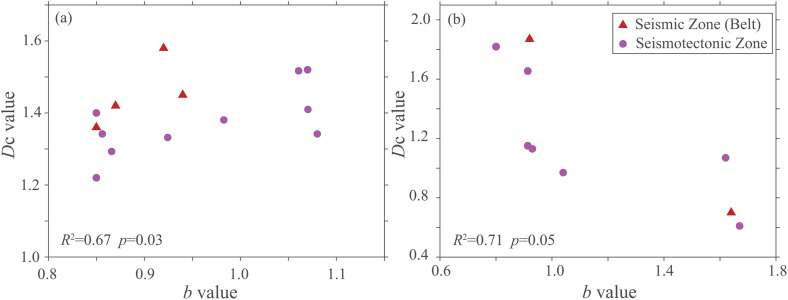
Spatial coupling relationship between fractal dimension (*D*_c_) and *b*-value. (a) Positive correlation regions (*R*^2^ = 0.67, *p* = 0.03), mainly distributed in moderate-value zones; (b) Negative correlation regions (*R*^2^ = 0.71, *p* = 0.05), mainly distributed in extreme-value zones.

To investigate the correlation between seismicity parameters and comprehensively assess regional seismic hazard in North China, this study focuses on the spatial coupling relationship between the fractal dimension of background seismicity and the *b*-value. The results reveal complex correlation characteristics between the two across the region. Overall, the spatial distributions of *D*_c_ and *b*-values show a discernible correlation (Fig 4). A significant negative correlation (*R*² = 0.71, *p* = 0.05) exists in areas with extreme fractal dimensions, where high *D*_c_ spatially coincides with low *b*-values, and vice versa. This indicates that even in fractured media, stress can localize along major faults. Consequently, while seismic activity may appear spatially diffuse overall, energy release tends to localize along dominant faults. Areas characterized by low *b*-values and high *D*_c_-values may therefore represent a background conducive to the preparation of larger earthquakes.

However, within regions where the fractal dimension falls between the first and third quartiles, a positive correlation emerges (*R*^2^ = 0.67, *p* = 0.03). This suggests that highly fragmented media lead to dispersed stress distribution, favoring the release of strain energy through numerous, spatially scattered small earthquakes, reflecting the complexity of tectonic and seismogenic patterns in North China. From a seismic hazard perspective, different parameter combinations reveal distinct risk profiles. The co-occurrence of low *b*-values (indicative of relatively more large earthquakes) and low fractal dimensions (indicating highly clustered seismicity) in the Yinchuan-Hetao seismic zone and lower Yangtze-South Yellow Sea seismic zone may signal high hazard due to stress concentration on major faults. Conversely, the combination of low *b*-values (suggesting high stress background) and high fractal dimensions (reflecting spatially diffuse seismicity) in the Fen-Wei and central-northern North China Plain seismic zones indicates a high-stress state coupled with complex fault networks. This complexity results in a highly disordered spatial distribution of seismicity while maintaining the potential for moderate-to-strong earthquakes. The relationship between fractal dimension and *b*-value is nonlinear. It exhibits a negative correlation in extreme value zones and a positive correlation in moderate value zones, reflecting distinct stress structure interactions that are informative for seismic hazard assessment.

### 3.4. Correlation analysis between fractal dimension and geophysical fields

**Fig 5:**
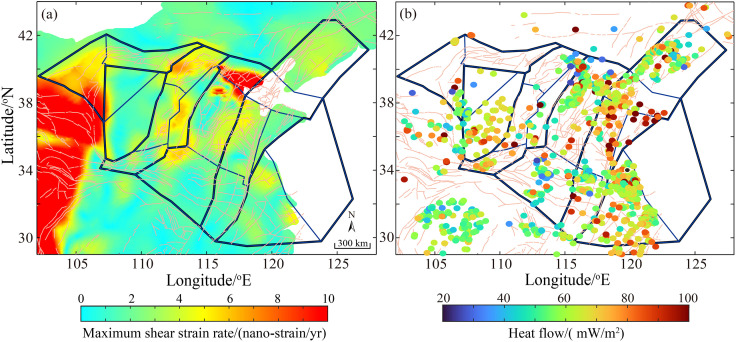
Spatial distribution of geophysical field characteristics in the North China Block. (a) Maximum shear strain rate (from Wang and Shen [53]) (b) Terrestrial heat flow (from Wang et al. [54]).

To objectively analyze the factors influencing the spatial fractal dimension of seismicity, we utilized maximum shear strain rate from Wang and Shen [53] and terrestrial heat flow compiled by Wang et al. [54] as the foundational datasets, their spatial distributions are shown in Fig 5. In the correlation analysis, parameters from the tectonically stable Ordos Block were excluded to focus on the response within active tectonic regions. To enhance the robustness of our conclusions and mitigate the influence of outliers, median values were used to represent both maximum shear strain rate and heat flow. The median is less sensitive to extreme values and better reflects the regional background level, thereby ensuring that the identified correlations are more robust and generalizable. Within the framework of seismic zoning, the spatial fractal dimension of seismicity was calculated for each grid cell. This value was then paired with the corresponding number of earthquakes (N), the seismicity parameter (*a*-value), the median maximum shear strain rate, and the median terrestrial heat flow within the same grid. The strength of the linear relationship between each parameter pair was quantified using Pearson’s correlation coefficient (*R*^2^).

**Fig 6:**
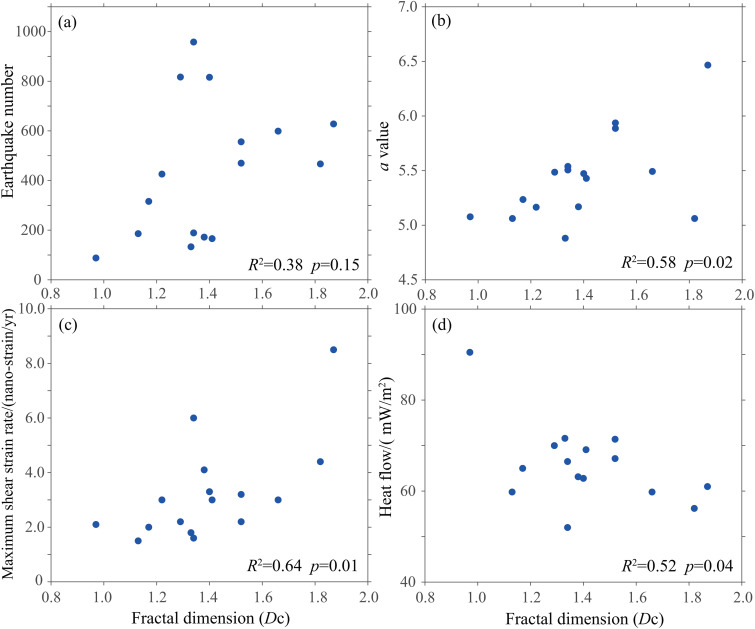
Quantitative relationships between fractal dimension (*D*_c_) and seismicity parameters/geophysical fields. Each data point represents a grid cell, *R*^2^ is the Pearson correlation coefficient, *p* is the significance levels. **(a)** *D*_c_ shows a weak positive correlation with earthquake count (*R*^2^ = 0.38, *p* = 0.15), indicating that event number is not the primary driver of *D*_c_ spatial variation. (b) *D*_c_ shows a moderate positive correlation with seismicity level *a*-value (*R*^2^ = 0.58, *p* = 0.02), suggesting that high-frequency seismic regions tend to have more complex spatial rupture networks. (c) *D*_c_ shows the strongest positive correlation with maximum shear strain rate (*R*^2^ = 0.64, *p* = 0.01), revealing that tectonic deformation intensity is the factor controlling seismic spatial complexity (d) *D*_c_ shows a negative correlation with terrestrial heat flow (*R*^2^ = 0.52, *p* = 0.04), revealing the modulating role of deep thermal state.

The fractal dimension shows only a weak positive correlation with the number of earthquakes (*R*^2^ = 0.38, *p* = 0.15, Fig 6a), suggesting that while a sufficient sample size is necessary for reliable calculation, the sheer number of events is not the primary physical driver of its spatial variation. A moderate positive correlation exists with the seismicity level (*R*^2^ = 0.58, *p* = 0.02, Fig 6b), indicating that regions with higher seismic frequency tend to have more complex spatial rupture networks. The strongest correlation is observed with tectonic deformation intensity, specifically the maximum shear strain rate (*R*^2^ = 0.64, *p* = 0.01, Fig 6c), implying that areas of higher crustal strain exhibit more complex and diffuse seismicity (higher fractal dimension). This identifies strain rate as the dominant controlling factor among those examined. Conversely, a negative correlation is found with terrestrial heat flow (*R*^2^ = 0.52, *p* = 0.04, Fig 6d). This contradicts the simplistic assumption that higher heat flow universally increases fracturing. Instead, it suggests that elevated heat flow may modify rock rheology, promote ductile deformation, or concentrate strain release along major faults, leading to more spatially clustered seismicity (lower fractal dimension). The size of the subregion shows no significant correlation with the number of earthquakes, which is primarily governed by tectonic activity. Consequently, the subregion area also lacks a clear relationship with the *b*-value and fractal dimension. The fractal dimension correlates positively with maximum shear strain rate (dominant driver) and negatively with terrestrial heat flow (modulator), revealing that tectonic forcing and deep thermal state jointly control seismic spatial complexity.

## 4. Discussion

The spatiotemporal evolution of the fractal dimension carries important dynamic information. Changes in the *b*-value and fractal dimension before major earthquakes [19] and their complex coupling reveal the tectonic complexity of the North China Block. The systematic variation in the fractal dimension around strong earthquakes reflects the evolution of the stress state and rupture process in the source region. The pre-seismic declining trend in fractal dimension may be closely related to the rupture nucleation process. The sharp drop in fractal dimension immediately after the mainshock reflects the high concentration of aftershocks on or near the main rupture surface. Although the temporal patterns observed around the Haicheng and Tangshan earthquakes align with theoretical expectations of strain localization, their universal applicability as a precursor requires validation across more events and regions.

The spatial fractal dimension and *b*-value exhibit a complex nonlinear relationship, showing negative correlation in extreme-value zones and positive correlation in moderate-value zones. A positive correlation may indicate aseismic slip or frequent small earthquakes that reduce the risk of large thrust events [29]. This reflects the coupled influence of medium heterogeneity and tectonic stress. Negative correlation in extreme zones suggests highly fractured media under high stress, leading to lower *b*-values and enhanced clustering (lower *D*_c_). Positive correlation in moderate zones implies a dynamic equilibrium between stress and structure, where spatial distribution and magnitude-frequency vary coherently. Different parameter combinations indicate distinct hazard states. The low *b*-value and low *D*_c_ combination, found at stable block margins or locked fault segments, reflects conditions favorable for strong earthquakes. Studies in the Himalaya confirm this pattern signals increased hazard [55]. The low *b*-value and high *D*_c_ combination, typical of fault intersections or structurally complex areas, indicates high stress but diffuse seismicity across multiple faults, thus also representing a high-risk setting [19,32]. These combinations improve hazard zoning by integrating spatial structure with traditional *b*-value analysis, enhancing the physical basis for long-term seismic assessment.

Correlation analysis between fractal dimension and geophysical parameters reveals multi-scale mechanisms controlling seismic spatial complexity. These interrelated relationships depict a cascading process from tectonic driving to local adjustment. Under sustained tectonic loading, crustal media form dense, interwoven fault networks, producing spatially diffuse and complex seismicity. This explains why higher fractal dimensions are typically observed along deformation fronts, such as major fault zones and block boundaries. The negative correlation between fractal dimension and heat flow challenges the intuitive notion that high heat flow promotes fracturing, instead revealing the profound influence of deep thermal processes. Rock mechanics studies bolster this interpretation by demonstrating that rising temperatures induce a transition from brittle to ductile behavior [56,57]. In high heat flow regions, the brittle-ductile transition occurs at shallower depths, confining seismogenesis to a thinner crustal layer and concentrating strain on major faults [58,59]. This mechanism explains why high heat flow areas exhibit more clustered seismicity (lower *D*_c_) despite high strain rates.

## 5. Conclusion

To thoroughly investigate the fractal structure of seismic activity in North China and its tectonic implications, this study applied the stochastic declustering method to process the earthquake catalog, effectively mitigating the clustering effects of earthquake sequences. Subsequently, the correlation dimension method and the maximum likelihood method were employed to systematically calculate the fractal dimension and seismicity parameters. The relationships among the fractal dimension, seismicity parameters, and geophysical field characteristics were then analyzed. The main conclusions are as follows:

(1) The application of the stochastic declustering method resulted in an increase in the spatial fractal dimension of background seismicity from 1.20 to 1.45, thereby effectively eliminating the influence of spatial clustering within earthquake sequences. Concurrently, the stability of the *b*-value before and after the declustering process confirms that this algorithm does not alter the inherent stress and medium properties it reflects.
(2) Seismic activity in North China exhibits distinct fractal characteristics. Areas with high fractal dimension (*D*_c_ > 1.52) are predominantly located in regions such as the Fen-Wei seismic zone and the central-northern segments of the North China Plain, where Cenozoic fault-depression basins intersect with complex fault zones, indicating highly fractured crustal media. In contrast, low-value areas correspond to tectonically more stable regions like the Ordos Block. The spatial distribution of the seismicity parameter *b*-value also shows significant heterogeneity.
(3) Analysis of the Tangshan and Haicheng earthquake cases reveals that the fractal dimension exhibited a characteristic pattern, a declining trend before the mainshocks, followed by a gradual post-seismic recovery. This temporal evolution closely correlates with strong seismic activity, suggesting it may reflect dynamic processes such as strain accumulation, rupture nucleation, and post-earthquake stress adjustment. These observations imply potential precursory significance for the fractal dimension.
(4) The spatial relationship between the fractal dimension and the *b*-value is complex, with a negative correlation in extreme-value zones and a positive correlation in moderate-value zones. This pattern reveals the coupled effects of media heterogeneity and stress state. Different parameter combinations (e.g., low *b*-value and low *D*_c_, low *b*-value & high *D*_c_) indicate distinct seismic hazard potentials, thereby providing more refined criteria for risk assessment.
(5) Our quantitative analysis demonstrates that the spatial fractal dimension correlates positively with maximum shear strain rate, indicating more dispersed seismicity in complex fault zones, and negatively with terrestrial heat flow, revealing the modulating role of deep thermal state. It also shows positive correlations with seismicity level (*a*-value) and event count. These findings collectively suggest that seismic spatial complexity is principally driven by tectonic dynamics, whereas its expression is modulated by the deep thermal regime.

This study deepens the understanding of the fractal characteristics of seismicity in North China and provides a new perspective for seismic hazard analysis. Moving forward, we may further combine high-resolution three-dimensional crustal models, rock mechanics experiments, and numerical simulations to clarify the microphysical mechanisms behind the spatiotemporal evolution of fractal dimensions, especially in relation to rupture nucleation, fluid interactions, and crustal rheological behavior.

Copyright: ©2026 Bi et al. This is an open access article distributed under the terms of the Creative Commons Attribution License, which permits unrestricted use, distribution, and reproduction in any medium, provided the original author and source are credited.

## Supporting information

S1 Table Data of Fig 1. Spatiotemporal characteristics of seismicity in the North China Block.
(XLS)

S2 Table Data of Fig 2. Temporal evolution of fractal dimension *D*_c_ and *b*-value before and after strong earthquakes.
(XLS)

S3 Table Data of Figs 3 and 4. Spatial distribution of the fractal dimension (*D*_c_) and the seismicity parameter *b*-value before and after declustering and Spatial coupling relationship between fractal dimension (*D*_c_) and *b*-value.
(XLS)

S4 Table Data of Fig 5. Spatial distribution of geophysical field characteristics in the North China Block.
(XLS)

S5 Table Data of Fig 6. Quantitative relationships between fractal dimension (*D*_c_) and seismicity parameters/geophysical fields.
(XLS)
